# 

**Published:** 1855-10

**Authors:** 


☞ Mr. A. Tilden is an authorized Agent for this Journal.

### PAYMENTS RECEIVED.

Dr. L. L. Ledbetter, Atlanta, Ga.; Drs. J. D. Keaton, W. T. Feay, Georgia; Dr. S. H. Harris, Mississippi; Drs. A. B. Wallace, W. C. Hanson, J. E. G. Terrell, D. B. Julian, C. R. Moore, M. G. Slaughter, G. W. Neely, R. M. Waldrop, J. J. Newsome, N. B. Drewry, A. M. Parker, Georgia.

### PRIVATE MEDICAL INSTRUCTION.

THE undersigned design to give a Course of Private Instruction to such Medical Students as may desire to enter their office. Regular lectures and examinations on the various branches of medical science will be afforded twice a week.

J. G. WESTMORELAND, M. D.,
Atlanta, Sept. 1, 1855. W. F. WESTMORELAND, M. D

